# Efficiency of Spermatogonial Dedifferentiation during Aging

**DOI:** 10.1371/journal.pone.0033635

**Published:** 2012-03-19

**Authors:** Chihunt Wong, D. Leanne Jones

**Affiliations:** 1 Laboratory of Genetics, The Salk Institute for Biological Studies, University of California San Diego, La Jolla, California, United States of America; 2 Division of Cell and Developmental Biology, Department of Biological Sciences, University of California San Diego, La Jolla, California, United States of America; National Cancer Institute, United States of America

## Abstract

**Background:**

Adult stem cells are critical for tissue homeostasis; therefore, the mechanisms utilized to maintain an adequate stem cell pool are important for the survival of an individual. In *Drosophila*, one mechanism utilized to replace lost germline stem cells (GSCs) is dedifferentiation of early progenitor cells. However, the average number of male GSCs decreases with age, suggesting that stem cell replacement may become compromised in older flies.

**Methodology/Principal Findings:**

Using a temperature sensitive allelic combination of *Stat92E* to control dedifferentiation, we found that germline dedifferentiation is remarkably efficient in older males; somatic cells are also effectively replaced. Surprisingly, although the number of somatic cyst cells also declines with age, the proliferation rate of early somatic cells, including cyst stem cells (CySCs) increases.

**Conclusions:**

These data indicate that defects in spermatogonial dedifferentiation are not likely to contribute significantly to an aging-related decline in GSCs. In addition, our findings highlight differences in the ways GSCs and CySCs age. Strategies to initiate or enhance the ability of endogenous, differentiating progenitor cells to replace lost stem cells could provide a powerful and novel strategy for maintaining tissue homeostasis and an alternative to tissue replacement therapy in older individuals.

## Introduction

In regenerative tissues, such as skin and blood, adult stem cells support tissue homeostasis by replenishing cells lost due to normal cellular turnover and/or damage throughout life. Stem cells are found in unique locations within a tissue, known as stem cell niches, which support stem cell self-renewal, maintenance, and survival. Stem cell self-renewal provides a means to maintain a pool of active stem cells; however, in some tissues, the number and/or activity of stem cells declines during aging, suggesting that changes in stem cell behavior likely contribute to reduced tissue homeostasis in older individuals (reviewed in [1]).

**Figure 1:**
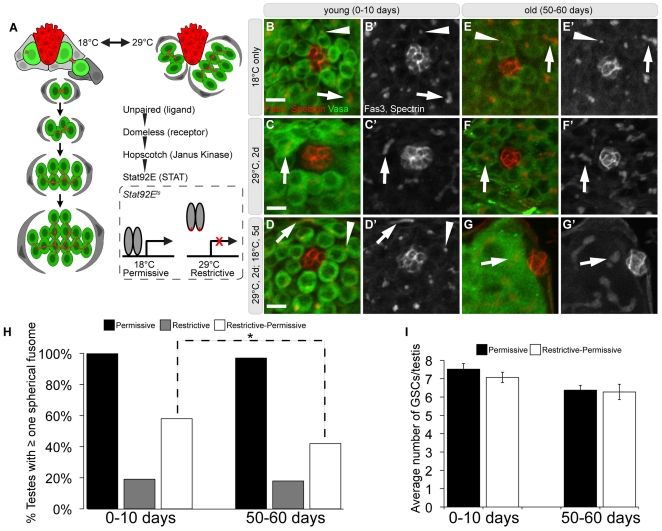
The effect of aging on germ line dedifferentiation in the *Drosophila* testis. (A) Schematic of early spermatogenesis and the *Stat92E^ts^* reversion paradigm. GSCs (light green) and cyst stem cells (CySCs, light gray) surround the hub (red). A pair of CySCs envelopes each GSC, while cyst cells (dark gray) envelope gonialblasts and spermatogonial cysts (dark green). Red dots and branched structures represent a germ cell-specific organelle called the fusome. Jak-STAT signaling is required for stem cell maintenance. In flies carrying the temperature sensitive allele of *Stat92E*, GSCs differentiate at the restrictive temperature (29°C). Upon recovery at the permissive temperature (18°C), spermatogonia revert to GSCs. (B–G′) Immunofluorescence images of testes stained for Fasciclin3 (Fas3) (hub; red), alpha-spectrin (fusomes; red), and Vasa (germ cells; green) during dedifferentiation. (B-B′,E-E′) Spectrosomes (arrowheads) within GSCs and branched fusomes (arrows) within spermatogonia at 18°C in young (B-B′) or aged (E-E′) flies. (C-C′,F-F′) Branched fusomes within spermatogonia next to the hub (arrows) in flies shifted to 29°C for 2 days in young (C-C′) or aged (F-F′) flies. (D-D′) Spectrosomes within revertant GSCs (arrowheads) adjacent to the hub in young flies shifted back to 18°C for 5 days. (G-G′) Spermatogonia that contact the hub contain branched fusomes in testes from aged flies shifted back to 18°C for 5 days. (H) Quantification of testes containing at least one spectrosome in young and aged flies at 18°C (Permissive), 29°C (Restrictive), and recovery at 18°C (Restrictive-Permissive). (I) Average number of GSCs in testes that contained GSCs in young and aged flies raised at 18°C and after the recovery at 18°C. Error bars represent standard error of the mean (SE). Bracket with * shows statistically significant changes, p<0.001 Scale bar: 10 µm. Genotype: *Stat92E^F^/Stat92E^06346^*.

In the *Drosophila* testis, male germline stem cells (GSCs) and cyst stem cells (CySCs) are located at the apical tip where they are in contact with a cluster of somatic cells called the hub (Figure 1A). Hub cells secrete the ligand Unpaired (Upd), which activates the Janus kinase - Signal Transducer and Activator of Transcription (Jak-STAT) signal transduction pathway within adjacent stem cells to regulate self-renewal, maintenance, and niche occupancy [2], [3], [4], [5], [6]. When a GSC divides, one daughter cell remains in contact with the hub and retains stem cell identity, while the other daughter cell is displaced away from the hub and initiates differentiation as a gonialblast (GB). GBs undergo four rounds of mitotic amplification divisions with incomplete cytokinesis to produce a cyst of 16 interconnected spermatogonia (reviewed in [7]). A pair of CySCs encapsulates each GSC, aids in regulating GSC self-renewal, and cyst cells derived from CySCs ensure differentiation of the developing spermatogonia [8], [9], [10]. In addition to the Jak-STAT pathway, number of other factors have been shown to influence stem cell behavior and the relationship between the germ line and the niche in the testis [11], [12], [13], [14], [15], [16], [17], [18], [19]. Therefore, successful spermatogenesis requires adequate signaling between hub cells, CySCs, and GSCs to coordinate proper functioning of each cell population and tissue homeostasis [4], [9], [10], [20], [21], [22].

The *Drosophila* germ line has provided an excellent system for investigating the relationship between organismal aging and age-related changes in stem cell behavior [23], [24], [25], [26], [27], [28], [29]. Aging results in a decline in spermatogenesis, which can be attributed, at least in part, to a significant decrease in the average number of GSCs that progress through the cell cycle more slowly [25], [28], [30]. Based on the predicted half-life of male GSCs, the testis should be depleted of stem cells by 50 days [28]. However, we have observed a reproducible 35% decrease in the average number of GSCs [25], suggesting that mechanisms must exist to replace lost stem cells over time.

Stem cells could divide symmetrically to replace lost stem cells and maintain full occupancy of the niche, as was demonstrated in *Drosophila*, where symmetric division of GSCs was observed to replace neighboring GSCs lost to differentiation [31], [32]. In addition, reversion (dedifferentiation) of differentiating germ cells back to a stem cell state has been shown to occur *in vivo* in the germ line of both *Drosophila* and mice after depletion of the endogenous stem cell pool [32], [33], [34], [35], [36]. Furthermore, using a system to permanently mark differentiating spermatogonia in the *Drosophila* testis, marked GSCs were found in increasing numbers in response to DNA damage and in aged animals, suggesting that individual stem cells can be replaced by spermatogonia over time [30].

By using a dedifferentiation paradigm in which only germ cell behavior is modified, Sheng et al. demonstrated up to 100% efficiency in dedifferentiation, providing strong evidence that somatic cyst cells play an integral role in the dedifferentiation process [37]. Based on a model where the hub signals to CySCs that then relay self-renewal signals to GSCs [4], [22], efficient coordination and signaling between these three cell types must be required for dedifferentiation to occur. However, aging results in a significant decline in the expression of *upd* in hub cells [25]. Consequently, decreased Jak-STAT signaling in older males could not only affect stem cell self-renewal, but might also impact dedifferentiation of spermatogonia to replace lost GSCs [25], [37]. Therefore, we have used the *Drosophila* male germ line to investigate the impact of aging on dedifferentiation and to compare the aging of germline and somatic stem cells residing within the same niche.

## Results

### Germ cell dedifferentiation occurs in young and old males

**Figure 2:**
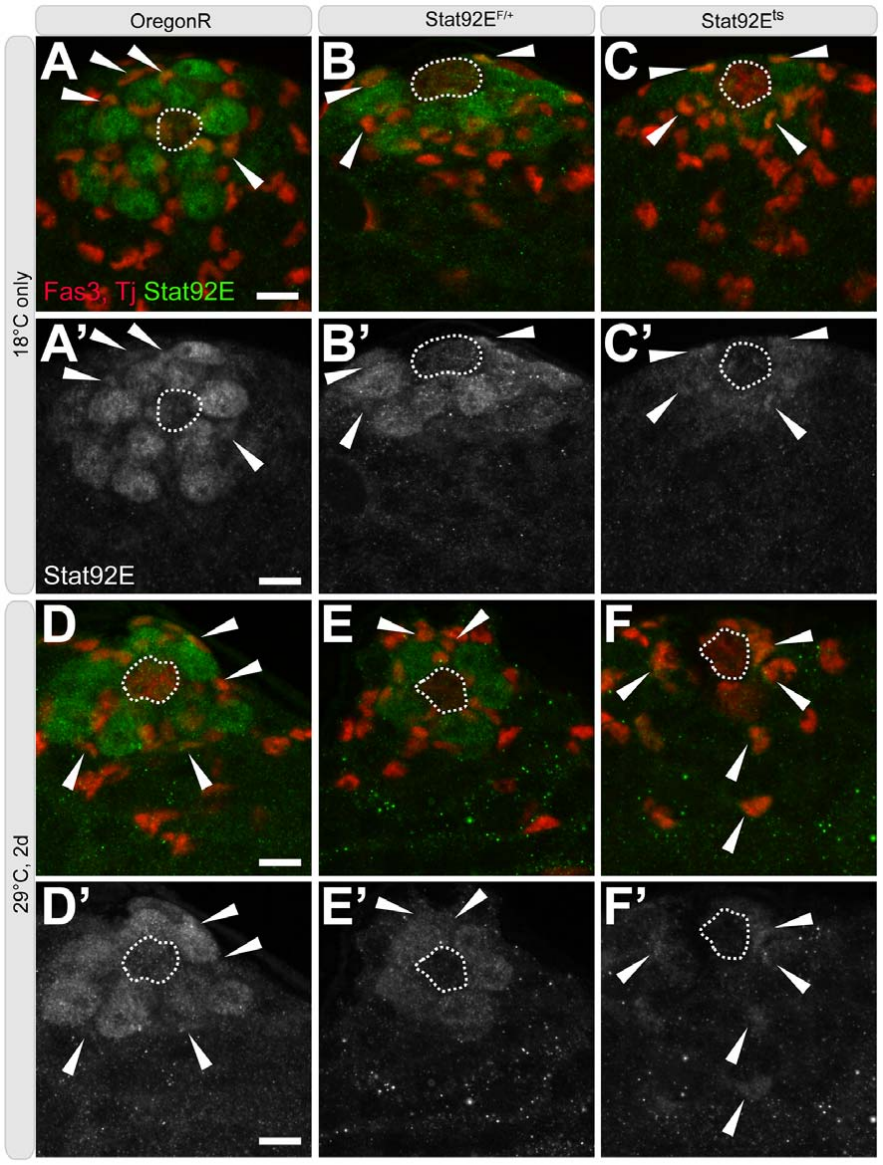
Stat92E localization in young flies during the reversion paradigm. (A–F′) Immunofluorescence images of testes from young OregonR, *Stat92E^F^*, and *Stat92E^ts^* flies stained for hub cells (Fas3, red, outline), early cyst cells (Traffic Jam [TJ], red), and Stat92E (green). (A–C′) at 18°C (D–F′) at 29°C for 2-days. Arrowheads point to Stat92E^+^TJ^+^ cells. Scale bars: 10 µm. Genotype: (A-A′,D-D′) *OregonR*; (B-B′,E-E′) *Stat92E^F^/*+; (C-C′) *Stat92E^F^/Stat92E^06346^*; (F-F′) *Stat92E^F^/Stat92E^J6C8^*.

To investigate the impact of aging on dedifferentiation, we used a previously characterized, temperature sensitive allelic combination of *Stat92E* (*Stat92E^ts^*) that permits manipulation of GSC differentiation within the niche (Figure 1A; see Materials and Methods) [34]. *Stat92E^ts^* (*Stat92E^F^/Stat92E^06346^*) or out-crossed, control (*Stat92E^F^*/+) flies were raised and aged at 18°C post-eclosion (hatching). To initiate dedifferentiation, flies were moved to the restrictive temperature (29°C) for two days, which leads to a decrease in detectable Stat92E protein in germ cells and a loss of GSCs due to differentiation (Figures 1, 2). Subsequently, the flies were shifted back to 18°C for five days and assayed for the ability of spermatogonia to replace lost GSCs.

Germ cell differentiation can be monitored by the morphology of the fusome, a germ cell-specific organelle that is spherical in GSCs and gonialblasts (spectrosome) and becomes progressively branched in spermatogonia during mitotic amplification divisions (Figure 1A). Therefore, to determine the efficiency of dedifferentiation, we quantified the number of testes with at least one spectrosome-containing germ cell at each point during the dedifferentation paradigm. At the permissive temperature (18°C), all testes from young *Stat92E^ts^* flies (0–5 days post-eclosion, dpe) contained at least one germ cell with a spectrosome adjacent to the hub (n = 46, Figure 1B-B′,H). When shifted to 29°C for two days, a significant decrease in the number of spectrosome-containing germ cells was observed in testes from *Stat92E^ts^* males (19%, n = 42; Figure 1C-C′,H), indicating that GSCs differentiated adjacent to the hub. However, when *Stat92E^ts^* flies are shifted back to 18°C, germ cells with spectrosomes were observed in 58% of testes examined (n = 101), verifying that spermatogonia had successfully dedifferentiated into single GSCs (Figure 1D-D′,H).

To determine whether aging affects the ability of spermatogonia to de-differentiate into stem cells, aged *Stat92E^ts^* males (50–55 dpe) were exposed to the dedifferentiation paradigm. Raising flies at lower temperatures slows development and delays the onset of aging phenotypes; thus, 50-day old males raised at 18°C resemble 30-day old males raised at 25°C. No difference was observed in the survival of *Stat92E^F/+^* out-crossed controls or *Stat92E^ts^* (*Stat92E^F/06346^* or *Stat92E^F/J6C8^*) mutant flies that were raised and maintained at 18°C for 50–60 days (data not shown).

In nearly all testes from aged *Stat92E^ts^* flies raised and maintained at 18°C, germ cells around the hub contained spectrosomes (97%, n = 55; Figure 1E-E′,H). When aged *Stat92E^ts^* flies were shifted to 29°C for 2 days, very few testes had spectrosome-containing germ cells adjacent to the hub (18%, n = 39; Figure 1F-F′,H), similar to the behavior of germ cells in young *Stat92E^ts^* males. However, the percentage of testes from aged, *Stat92E^ts^* flies with at least one spherical spectrosome was significantly reduced (42%, n = 120) when flies were shifted back to 18°C (Figure 1G-G′,H). This decrease is statistically significant when compared with the number of GSCs in testes from young *Stat92E^ts^* flies; however, this likely reflects the overall decrease in GSCs in older males [25], [28]. In testes where successful dedifferentiation had occurred, the average number of GSCs was comparable to that of age-matched controls that had been maintained at 18°C (Figure 1I). Similar results were obtained with another temperature sensitive combination of *Stat92E* alleles (Figure S1; Materials and Methods). Taken together, our observations indicate that germ cell dedifferentiation appears to be remarkably efficient in both young and aged males.

### Early cyst cell behavior during aging

**Figure 3:**
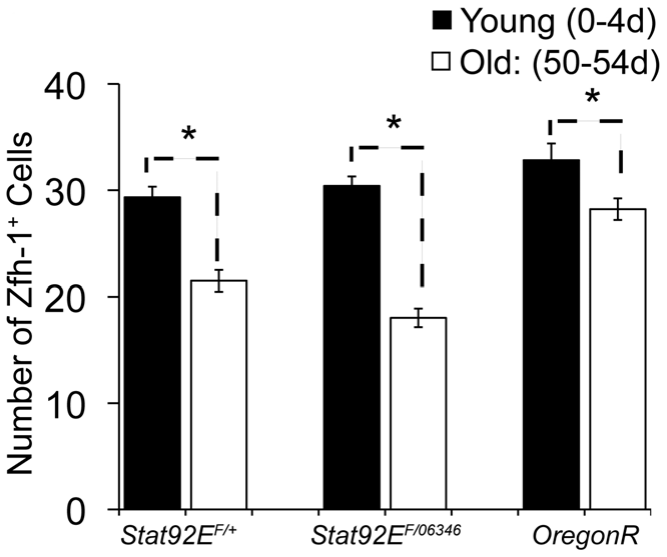
Decline in early cyst cells during aging. (A) Graph of the average number of Zfh-1^+^ cells in young and aged flies maintained at 18°C. Error bars represent standard error of the mean (SE). Genotypes: *Stat92E^F^*/+ , *Stat92E^F^/Stat92E^06346^* and OregonR.

Communication between germ cells and somatic cyst cells is essential not only for regulating GSC proliferation and the normal differentiation of spermatogonia, but it is also required for germline dedifferentiation [37]. Therefore, we wanted to assess the effect of aging on the number and activity of CySCs and early cyst cells, which surround spermatogonia undergoing mitotic amplification divisions. To quantify early cyst cell numbers in young and old flies, we stained for Zfh-1, a transcription factor expressed in cyst cells, including CySCs. The average number of Zfh-1^+^ cells decreased from 29.3 in testes from young flies to 21.5 in testes from 50-day old control males raised at 18°C (Figure 3; Table 1), consistent with recent results [38]. In *Stat92E^ts^* males maintained at 18°C, a similar aging-related decline in the average number of early cyst cells was observed (Figure 3; Table 1).

**Table 1:** Average number of Zfh-1^+^ cells in testes from young and aged males and during dedifferentiation.

Average Zfh1^+^		0–10d				50–60d			
Genotype	Temperature	Average	±	S.E.*	n	Average	±	S.E.	n
F/+	18°C only	29.3	**±**	0.989978273	27	21.5	**±**	1.016403242	41
	29°C 2d	34.5	**±**	0.975156271	33	22.3	**±**	0.673513554	44
	29°C 2d; 18c 1d	33.2	**±**	1.059794061	33	22.6	**±**	0.787992222	37
	29°C 2d; 18c 2d	27.9	**±**	0.792849044	49	21.9	**±**	0.760115801	34
			**±**				**±**		
F/06346	18°C only	30.4	**±**	0.88739882	37	18.0	**±**	0.870484051	39
	29°C 2d	22.5	**±**	1.025797512	30	13.4	**±**	1.096233896	37
	29°C 2d; 18c 1d	25.9	**±**	1.422814113	25	16.0	**±**	1.843554396	18
	29°C 2d; 18c 2d	29.3	**±**	1.229727291	54	17.6	**±**	1.47733466	19
			**±**				**±**		
F/J6C8	18°C only	25.7	**±**	0.87988033	43	18.5	**±**	0.928146507	30
	29°C 2d	18.8	**±**	0.770466843	39	15.6	**±**	0.648948915	50
	29°C 2d; 18c 1d	21.6	**±**	0.933421568	39	16.7	**±**	1.053799709	36
	29°C 2d; 18c 2d	24.4	**±**	0.757518756	54	18.5	**±**	0.88358523	42

* -Standard Error of the Mean.

**Figure 4:**
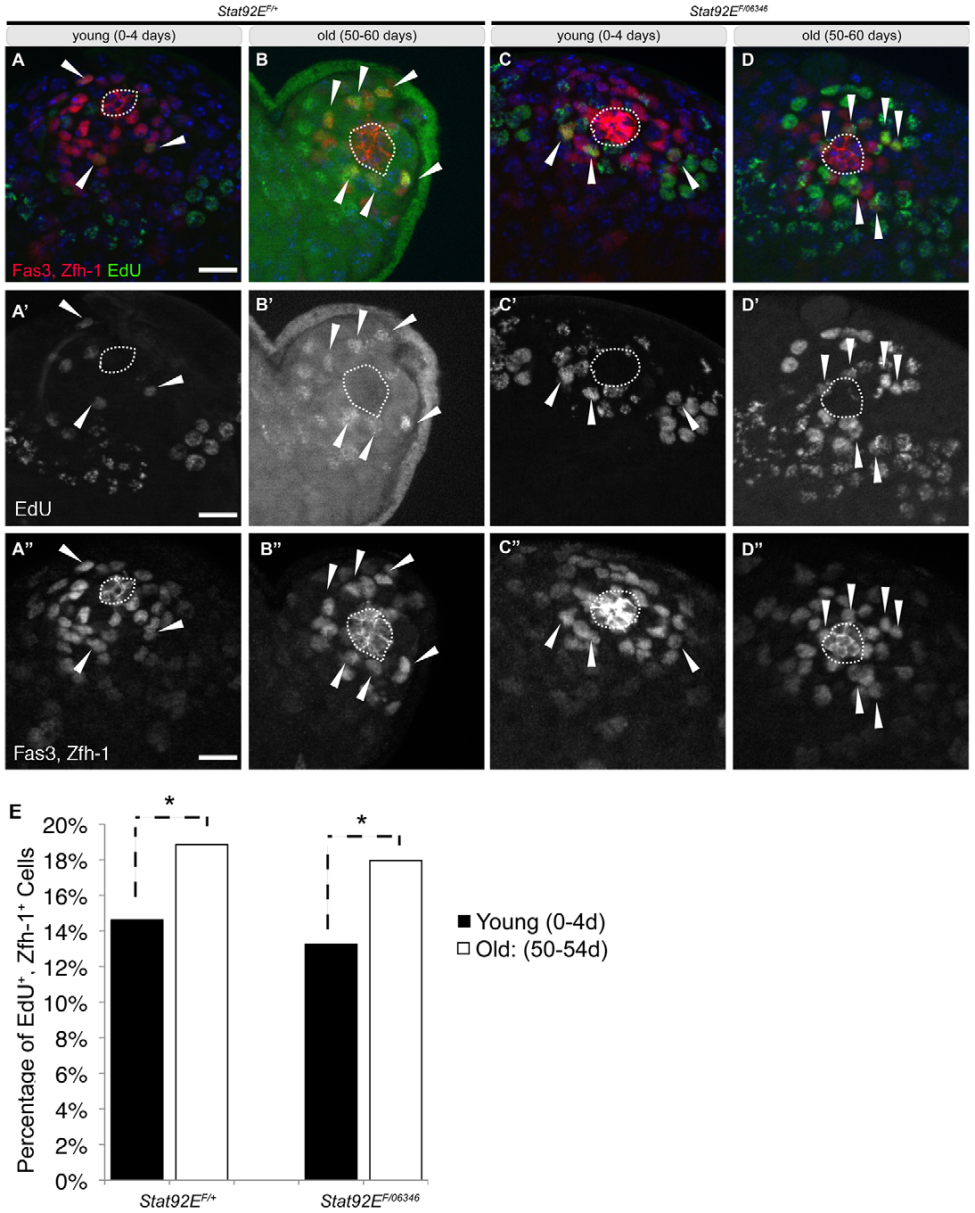
Increased proliferation of early cyst cells during aging. (A–D″) Immunofluorescence images of testes from young (A-A″; C-C″) and aged (B-B″; D-D″) flies stained for the hub (Fas3; red, outline), early cyst cells (Zfh-1; red) and labeled with EdU (green). Note EdU+, Zfh-1^+^ cells (arrowheads). Two merged 1 µm z-slices to represent majority of Zfh-1^+^ cells. (E) Graph of the percentage of Zfh-1^+^ cells in S-phase labeled by EdU in young and aged flies. Bracket with * shows statistically significant changes p<0.001. Scale bar: 10 µm. Genotype: *Stat92E^F^/^+^* and *Stat92E^ts^* (*Stat92E^F^/Stat92E^06346^*).

In addition to a decrease in the average number of GSCs with age, we and others observed a decrease in the proliferation rate of GSCs in older flies [25], [30]. Therefore, we also wanted to assay the effect of aging on the activity of CySCs. Although no specific markers for CySCs have been characterized, lineage tracing has placed them adjacent to the hub, intercalated between GSCs [39] where the vast majority of proliferative somatic cells reside [40], [41]. Therefore, quantifying the percentage of early cyst cells that are proliferating provides an indirect read-out for CySC number and activity. The proliferation rate of early cyst cells was determined using *ex vivo* incorporation of EdU, a thymidine analog, to label cells in S-phase, and the percentage of Zfh-1^+^ cells that were also EdU^+^ was calculated (S-phase index). The S-phase index of early cyst cells was 14.6% in testes from young control (*Stat92E^F^/+*) males raised at 18°C, which increased to 18.9% when control males were aged for 50 days at 18°C (Figure 4; Table 2). An increase in the S-phase index was also observed in testes from *Stat92E^ts^* males from 13.2% in 1-day old flies to 18% in 50-day old flies raised at 18°C (Figure 4; Table 2). Similar results were obtained with another temperature sensitive combination of *Stat92E* alleles (Figure S1). Therefore, in contrast to the decrease in proliferation observed in GSCs with age, CySCs, as a population, appear to become more active over time.

**Table 2:** S-phase index for cyst stem cells in testes from young and aged males and during dedifferentiation.

S-phase index		0–10d		50–60d	
Genotype	Temperature	S-Phase Index	n	S-Phase Index	n
F/+	18°C only	15%	29	19%	25
	29°C 2d	11%	33	10%	44
	29°C 2d; 18c 1d	17%	33	14%	37
	29°C 2d; 18c 2d	15%	49	20%	34
F/06346	18°C only	13%	24	18%	21
	29°C 2d	9%	30	13%	37
	29°C 2d; 18c 1d	13%	25	14%	18
	29°C 2d; 18c 2d	9%	54	15%	19
F/J6C8	18°C only	18%	23	23%	21
	29°C 2d	8%	39	10%	50
	29°C 2d; 18c 1d	14%	39	12%	36
	29°C 2d; 18c 2d	13%	54	15%	42

### Early cyst cell behavior during dedifferentiation

**Figure 5:**
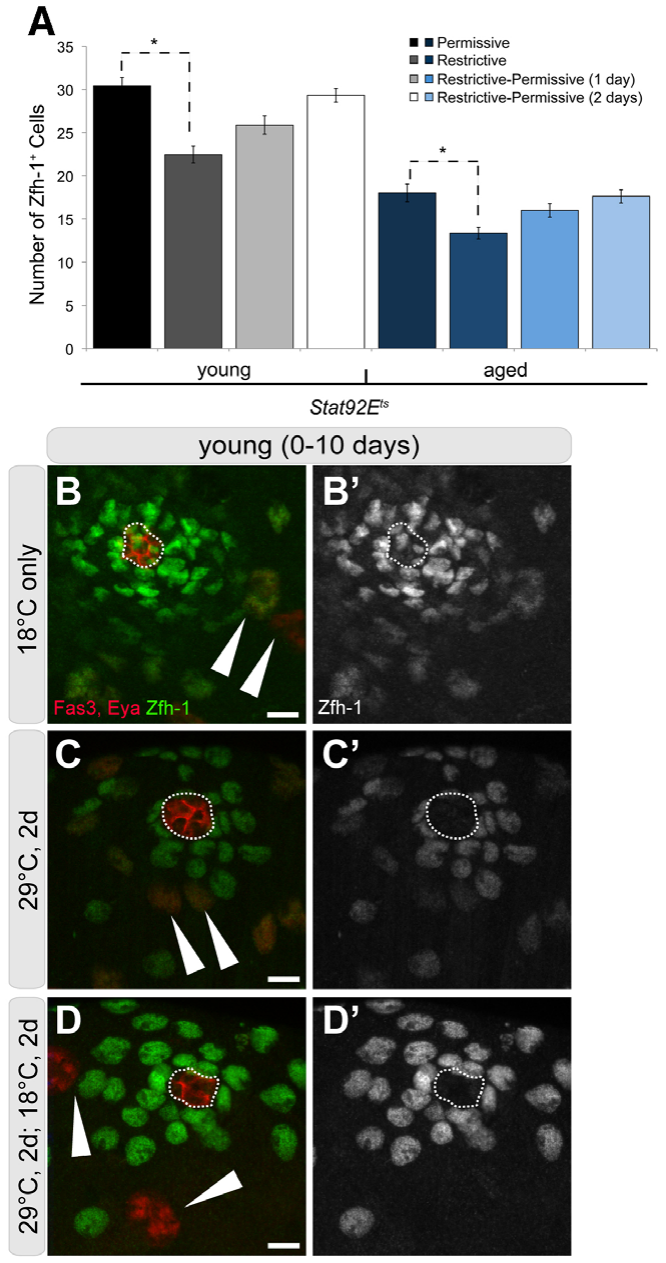
Behavior of early cyst cells during dedifferentiation. (A) Graph of the average number of Zfh-1^+^ cells throughout the reversion paradigm in young and aged, *Stat92E^ts^* flies. Error bars represent standard error of the mean (SE). Bracket with * shows statistically significant changes p<0.001. Genotype: *Stat92E^ts^* (*Stat92E^F^/Stat92E^06346^*). (B–D′) Immunofluorescence images of testes from young flies throughout the reversion paradigm stained for the hub (Fas3; red, outline), late cyst cells (Eyes Absent [EyA]; red, arrowhead), and early cyst cells (Zfh-1; green). (B-B′) at 18°C, (C-C′) at 29°C for 2 days, and (D-D′) recovery at 18°C for another 2 days. Number of merged 1 µm z-slices to represent majority of Zfh-1^+^ cells for (A-A′) z = 4 (B-B′) z = 1 (C-C′) z = 2. Note changes in density of Zfh-1^+^ cells. Scale bars: 10 µm. Genotype: *Stat92E^F^/Stat92E^06346^*.

Given the intimate relationship between GSCs and CySCs, we hypothesized that the sustained activity of early cyst cells could play an important role in facilitating spermatogonial dedifferentiation in older animals. Therefore, we wanted to assay the behavior of early cyst stem cells during dedifferentiation in young and aged males. In young *Stat92E^ts^* males, the average number of Zfh-1^+^ cells decreased to 22.5 (Figure 5A; Table 1) at 29°C, demonstrating that cyst cells are sensitive to a decline in Stat92E activity. After a 1-day recovery at 18°C, the number of Zfh-1^+^ cells increased to 25.9 (n = 25) and was fully restored to 29.3 (n = 54) after just 2 days of recovery at 18°C (Figure 5; Table 1). Interestingly, Stat^+^ somatic cells were visible, despite the decrease in Stat^+^ GSCs, indicative of residual Stat92E activity in somatic cells in this genetic background (Figure 2).

Although the average number of Zfh-1^+^ cells was lower in older *Stat92E^ts^* males (Figure 3), a similar trend was observed during the dedifferentiation paradigm. Shifting to 29°C for two days resulted in a drop in Zfh-1^+^ cells to 13.2 (n = 37); however, the number increased to 16.0 (n = 25) after only one day of recovery, and a complete restoration to 17.6 (n = 19) was observed after 2 days of recovery at 18°C (Figure 5A; Table 1). Similar results were obtained with another temperature sensitive combination of Stat92E alleles (Figure S1), suggesting that early cyst cells are rapidly restored in both young and aged flies under the dedifferentiation paradigm. Importantly, no decrease in the average number of Zfh-1^+^ cells was detected in young or aged, control (*Stat92E^F^/+*) flies shifted to 29°C (Table 1), indicating that high temperature does not affect early cyst cell number.

One explanation for a decrease in Zfh-1^+^ cells at the non-permissive temperature could be that the cyst cells are differentiating in concert with the germ line. Cyst cells that surround differentiating spermatocytes express the transcription factor Eyes Absent (Eya); however, no Eya^+^ cells were observed adjacent to the hub in young *Stat92E^ts^* males shifted to 29°C for 2 days (Figure 5 B-D′). Therefore, the decrease in Zfh-1^+^ cells does not appear to be due to direct differentiation into Eya^+^ cells, which is consistent with previous observations [37].

We next determined the S-phase index of early cyst cells after young *Stat92E^ts^* males were allowed to recover for one day at 18°C, prior to complete replacement of Zfh-1^+^ cells. The S-phase index for early cyst cells in young *Stat92E^ts^* males returned to baseline from 9.1% at 29°C (n = 30) to 13.5% upon recovery at 18°C (n = 25) (Table 2). This resumption of proliferation is also observed in young and aged control (*Stat92E^F^/+*) males after two days of recovery at 18°C (Table 2). Surprisingly, the S-phase index of early cyst cells in aged *Stat92E^ts^* males remained relatively stable at 29°C (13.3%; n = 37) when compared to their activity at 18°C (13.9%; n = 18) (Table 2). Similar results were obtained with another temperature sensitive combination of Stat92E alleles (Table 2).

In conclusion, a decrease in the complement of Zfh-1^+^ early cyst cells occurs during dedifferentiation in both young and aged males, which is accompanied by a decrease in the S-phase index of early somatic cells. The activity of Zfh-1^+^ cells quickly returns to baseline when flies are allowed to recover at the permissive temperature, followed subsequently by recovery of early cyst cell numbers. Similar trends are observed in young and aged males; however, the proliferation of early cyst cells, which is higher in aged males (Table 2), appears to remain relatively constant throughout the dedifferentiation paradigm. Maintenance of cyst cell activity is consistent with the presence of Stat^+^ cyst cells, even at the non-permissive temperature (Figure 2).

## Discussion

Several mechanisms have been described that are utilized to replace lost stem cells, including symmetric divisions and dedifferentiation of progenitor cells [31], [32], [33], [34], and studies have indicated that dedifferentiation contributes to replacement of male GSCs throughout life [30]. Here we demonstrate that dedifferentiation remains surprisingly robust in testes from aging flies, despite an overall decrease in GSCs (Figure 1I). Although it is known that the Jak-STAT pathway regulates the process of dedifferentiation in the *Drosophila* testis, little is known about the mechanisms by which spermatogonia are recruited into the niche and are able to reassume asymmetric, self-renewing divisions [37]. Regardless, it is clear that the somatic cyst cells play an active role in regulating the dedifferentiation process [37].

We have assayed the effects of aging on somatic cells within the *Drosophila* testis and found that despite an age-related decrease in early cyst cell numbers, those cyst cells that remain are more active as revealed by an increased percentage of early somatic cells that are progressing through S-phase (Figure 4; Tables 1, 2). The increase in cyst cell activity is reminiscent of the behavior of somatic cells in the complete absence of germ cells, as in the case of agametic animals [39], which may reflect a decline in anti-proliferative signals normally emanating from germ cells. Alternatively, the increase in early cyst cell activity with age may represent a mechanism by remaining cyst cells to compensate for the age-related loss of somatic cells.

In addition, we found that somatic cell number and activity recovers rapidly after dedifferentiation, providing a pool of somatic cells to facilitate spermatogonial dedifferentiation (Figure 5, Tables 1 and 2). Given the important role that cyst cells play in facilitating spermatogonial dedifferentiation and the fact that somatic cells appear to remain quite active in older animals (Figure 4, Tables 1 and 2), we propose that this could be one reason dedifferentiation remains robust during aging.

Dedifferentiation is a conserved process that has been long appreciated as a component of regeneration of tissues in amphibians and fish [42]; however, dedifferentiation as a potential mechanism for maintenance or repair of mammalian tissues has not been fully explored. A better understanding of how dedifferentiation of progenitor cells can replace lost or damaged stem cells could provide insight into this process. Future studies that characterize specific stem cell markers and signaling pathways, and the development of technologies that allow long-term *in vivo* imaging of tissues undergoing regeneration will be critical for understanding the physical interplay between stem and support cells within the context of a shared niche. Furthermore, the mechanisms regulating the reversion of a germ cell to a GSC may shed light on the plasticity of differentiated cell types and their conversion to induced pluripotent stem cells (iPS cells) to be used for disease modeling and stem cell-based therapies. Ultimately, strategies to initiate or enhance the ability of endogenous, differentiating progenitor cells to replace lost stem cells could provide a powerful alternative to stem cell transplantation and tissue replacement therapy in older individuals.

## Materials and Methods

### Fly husbandry and stocks

Flies were raised on standard cornmeal-molasses-agar medium at 18°C unless otherwise indicated. Newly eclosed 0–5 day old male flies were collected in vials containing up to 30 males and 10 females. Vials for aging experiments were supplemented with fresh yeast paste and changed every 7 days.

Three *Stat92E* mutant alleles were used: temperature sensitive mutant allele *Stat92E^Frankenstein^* (*Stat92E^F^*) (gift from E. Matunis) [43], the null *Stat92E^06346^* (Bloomington) [44], and *Stat92E^J6C8^* alleles (gift from N. Perrimon) [45]. The temperature sensitive heteroalleic combinations (‘Stat92E^ts^’) used were: *Stat92E^F^/Stat92E^06346^* and *Stat92E^F^/Stat92E^J6C8^*, and specific genotypes are noted in figure legends. ‘Control’ flies are *Stat92E^F^*/^+^ (*Stat92E^F^* out-crossed to wildtype OregonR females). For reversion assays, flies were raised at 18°C, shifted to 29°C for 2 days, shifted back to 18°C and allowed to recover for 5 days before assaying for GSC reversion [34], or for 1 or 2 days for early cyst cell recovery.

### Immunofluorescence and Microscopy

Immunofluorescence was performed as described previously [25]. The polyclonal rabbit anti-Vasa (1∶3000, gift from P. Lasko), guinea pig anti-Traffic Jam (1∶3000) (gift from D. Godt), rabbit anti-Stat92E (1∶800)(gift from D. Montell) and guinea pig anti-Zfh1 (1∶3000) (gift from C. Doe) were used at indicated concentrations. Mouse anti-alpha-spectrin (3A9)(1∶10), mouse anti-Fasciclin3 (7G10)(1∶10), and mouse anti-Eya (EYA10H6)(1∶10) were obtained from the Developmental Studies Hybridoma Bank (developed under the auspices of the National Institute of Child Health and Human Development and maintained by The University of Iowa, Department of Biological Sciences). Secondary antibodies were obtained from Molecular Probes. Samples were mounted in Vectashield mounting medium with 4′,6-diamidino-2-phenylindole (DAPI) (Vector Laboratories). Images were obtained using a Zeiss Axiovert 200 M or a Zeiss AxioObserver and processed using AxioVision (version 4.8; Carl Zeiss) and Adobe Photoshop software (Mountain View, CA).

### Quantification of average number of GSCs and the percentage of testes containing spectrosomes

GSCs are defined as a germ cell (Vasa^+^) that contacts Fasciclin 3^+^ hub cells. Only those samples with an easily distinguishable hub within the sizes of 10–18 µm were included in the quantification. Testes were counted as containing a spectrosome when at least one GSC contained a spectrosome marked by anti-alpha-spectrin. A Chi-squared test was performed to evaluate statistical significance. A Student's two-tailed *t*-test was performed to evaluate statistical significance of GSC counts after dedifferentiation. Above experiments were performed at least 2 times with a total n≥40 testes for percentage of spherical fusomes or n≥30 for average number of GSCs. Standard error of the mean values were calculated for averages.

### Ex Vivo EdU incorporation

EdU incorporation was performed and analyzed using the Click-iT® EdU Imaging Kit (Invitrogen), with the following modifications. All procedures were performed at room temperature with minimal exposure to light. Crude dissection of testes was performed in 1× Ringer's buffer and then transferred immediately to 1× Ringer's buffer in a glass embryo dish for no more than 10 minutes. Testes were subsequently transferred to 30 µM EdU diluted in 1× Ringer's buffer for 30′. After incorporation, testes were fixed for 20′ in 4% paraformaldehyde diluted in 1× PBS, followed by two washes with 1× PBST (0.5% Triton-×100) and blocked with 3% BSA in 1× PBS. Testes were bathed in the Click-iT® reaction cocktail for 30 minutes. IF was performed as indicated above.

### Quantification of average number of Zfh-1^+^ early cyst cells and Percentage of EdU+ cells

All Zfh-1^+^ early cyst cells around the hub were counted, unless they exceeded 30 µm away from the hub. A Student's two-tailed *t*-test was performed to evaluate statistical significance (p<0.05). An EdU^+^ cell was assayed as positive when the majority of the cell was labeled and EdU^+^ co-localized with Zfh-1. The S-phase index was determined as the number of EdU^+^ cells/Zfh-1^+^ cell X 100. A Tukey-Kramer HSD test was used to determine statistical significance, (positive value was considered significant). Experiments were performed at least 2 times with a total n≥30 testes, unless otherwise stated in text. Standard error of the mean values were calculated for averages.

## Supporting Information

Figure S1 **The effect of aging on germ line dedifferentiation in the** ***Drosophila*** **testis.** (A) Graph of the percentage of testes containing at least one spectrosome in young and aged flies throughout the reversion paradigm at 18°C (Permissive), 29°C for 2 days (Restrictive), and shift back to 18°C for 5 days (Restrictive-Permissive). (B) Graph of the average number of GSCs before and after the reversion paradigm. Graph of the average number of Zfh-1^+^ cells throughout the reversion paradigm in young and aged flies. Error bars represent standard error of the mean (SE). (D) Graph of the percentage of Zfh-1^+^ cells in S-phase labeled by EdU in young and aged flies. Error bars represent standard error of the mean (SE). Bracket with * shows statistically significant changes. p<0.001 Genotype: *Stat92E^F^/Stat92E^J6C8^*.
(TIF)
